# The complete mitochondrial genome and phylogenetic analysis of *Acanthochitona rubrolineatus* (Lischke, 1873)

**DOI:** 10.1080/23802359.2019.1642159

**Published:** 2019-07-17

**Authors:** Xiaoyu Guo, Yutong Cui, Shanshan Wang, Yanran Xu, Xiaoyue Sun, Ruoran Li, Yunhui Wang, Jiangyong Qu, Xumin Wang, Xiumei Liu

**Affiliations:** College of Life Sciences, Yantai University, Yantai, China

**Keywords:** *Acanthochitona rubrolineatus*, mitochondrial genome, phylogenetic analysis

## Abstract

We describe the complete mitochondrial genome of the important Polyplacophora species, *Acanthochitona rubrolineatus*. The mitogenome sequence of *A. rubrolineatus* is 14,988 bp, and all genes show the typical gene arrangement conforming to the Mollusca consensus. The overall base composition of the genome is T 39.0%, C 12.4%, A 31.2% and G 17.4%. The cytochrome c oxidase subunit I (COI) sequence of *A. rubrolineatus* and other 111 species from Chitonida were used for phylogenetic analysis by Bayesian inference and maximum-likelihood methods. The results show that *A*. *rubrolineatus*, *Acanthochitona achates*, and *Acanthochitona defilippi* is sister group to three lineages, and both together as the sister group of *A*. *rubrolineata*.

## Introduction

*Acanthochitona rubrolineatus* (Lischke, 1873) (Polyplacophora, Neoloricata, Cryptoplacidae) play an important role in the marine ecosystem and are widely distributed along the China Sea coast. At present, *A. rubrolineatus* was mostly used for the properties of biogenic magnetite nanoparticles in the radula and the traditional Chinese medicine. The population genetic diversity of *A. rubrolineatus* is threatened by coastal land reclamation and commercial exploitation so that it is urgent to perform genetics investigatio*n on A. rubrolineatus*.

In this study, *A. rubrolineatus* was collected from Laizhou, Shandong Province, China (N 37.18°, E 119.93°), and stored in 100% ethanol immediately after collection and then preserved in a refrigerator at −80 °C (Pu et al. 2017). The specimen was deposited in the marine specimen room of Yantai University with an accession number YTU-SKY-20181004. The genomic DNA was extracted from the muscle tissue by DNeasy Blood & Tissue Kit (Qiagen, Hilden, Germany) in accordance with the manufacturer’s protocol. The mitochondrial genome sequences were analyzed in the Illumina Hiseq 4000 sequencing system (Illumina, San Diego, CA) and annotated by the mitochondrial genome annotation server (Bernt et al. 2013) and tRNAscan-SE server (Lowe and Chan 2016).

The complete mitogenome sequence of *A. rubrolineatus* is a closed-circular molecule of 14,988 bp in length (GenBank accession no. KY827039) and contains 13 protein-coding genes (PCGs), 22 transfer RNA (tRNA) genes, and 2 ribosomal RNA (rRNA) genes. All genes show the typical gene arrangement conforming to the Mollusca consensus. The overall base composition of the genome is T 39.0%, C 12.4%, A 31.2%, and G 17.4%, exhibiting an A + T bias (70.2%). Among the 13 PCGs of *A. rubrolineatus*, *COX1*, *COX2*, *ATP8*, *ATP6*, *NAD4L*, and *NAD3* use the typical ATG start codon, the rest seven genes use ATA as the initiation codon. The ten PCGs (*COX1*, *COX2*, *ATP8*, *ATP6*, *NAD4L*, *Cytb*, *NAD6*, *NAD1*, *NAD3*, and *NAD2*) use TAA as the stop codon and three PCGs (*NAD5*, *NAD4*, and *COX3*) end with TAG. All tRNAs have the typical cloverleaf structure ranged from 57–68 bp.

**Figure 1.**
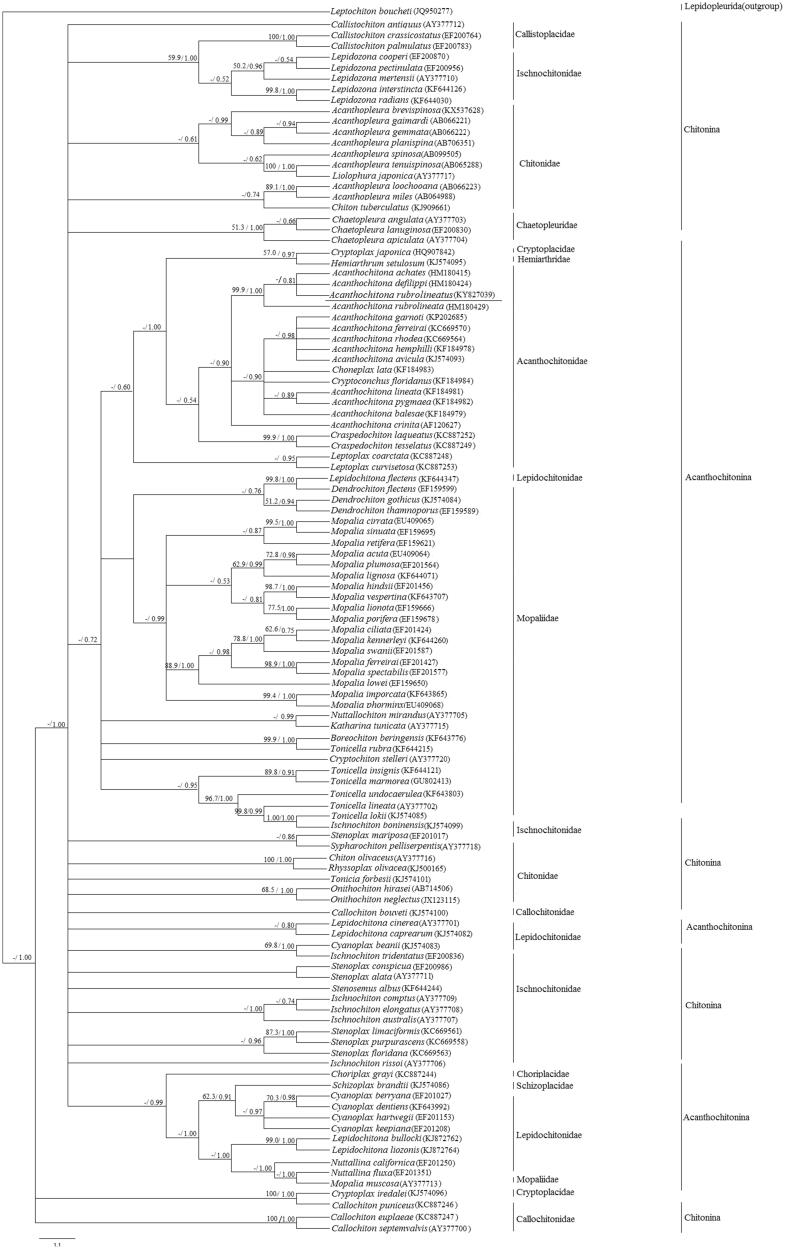
Bayesian tree of Chitonida was constructed by cytochrome c oxidase subunit I. ML shows the similar topological structure with BI. The bootstrap values for the BI and ML analysis were shown on the nodes (left is ML bootstrap values, right is BI bootstrap values, ‘-’ means the bootstrap values less than 50%). The underlined markers represent the species *Acanthochitona rubrolineatus* in this study. The brackets after the species mean Accession number from GenBank.

Bayesian inference (BI) (Huelsenbeck and Ronquist 2001) and maximum-likelihood (ML) (Guindon et al. 2009) methods were used to construct the phylogenetic structure by the cytochrome c oxidase subunit I (COI) sequence of *A. rubrolineatus* and other 111 species from the order Chitonida. For the data set, both BI and ML arrived at similar tree topologies for phylogenetic analyses (Figure 1). *Acanthochitona rubrolineatus* is recovered basal within Acanthochitonidae, receiving high support from BI (Figure 1). *Acanthochitona rubrolineatus*, *Acanthochitona achates*, and *Acanthochitona defilippi* form the sister group to three lineages, and both together are the sister group of *A*. *rubrolineata*, receiving high support from both ML and BI (Figure 1). It is obvious that there is no definite branch between the two suborders (Acanthochitonina, Chitonina), and the taxonomic status of the order Chitonida should be further evaluated.
